# Pragmatic trial of multifaceted intervention (STROKE-CARD care) to reduce cardiovascular risk and improve quality-of-life after ischaemic stroke and transient ischaemic attack –study protocol

**DOI:** 10.1186/s12883-018-1185-2

**Published:** 2018-11-06

**Authors:** Thomas Toell, Christian Boehme, Lukas Mayer, Stefan Krebs, Clemens Lang, Karin Willeit, Barbara Prantl, Michael Knoflach, Gerhard Rumpold, Gudrun Schoenherr, Andrea Griesmacher, Peter Willeit, Julia Ferrari, Wilfried Lang, Stefan Kiechl, Johann Willeit

**Affiliations:** 1 Department of Neurology, Medical University of Innsbruck, Anichstraße 35, A-6020 Innsbruck, Austria; 2 Department of Neurology, Hospital St. John of God, Johannes von Gott Platz 1, A-1020 Vienna, Austria; 3 Department of Neurology, Inselspital Bern, University Hospital, Freiburgstrasse, CH-3010 Bern, Switzerland; 4 Department of Medical Psychology, Medical University of Innsbruck, Schöpfstraße 23a, A-6020 Innsbruck, Austria; 5 Central Institute of Medical and Chemical Laboratory Diagnostics, University Hospital of Innsbruck, Anichstraße 35, A-6020 Innsbruck, Austria; 6 Sigmund Freud Private University, Medical Faculty, Campus Prater Freudplatz 1, A-1020 Vienna, Austria

**Keywords:** Stroke, Transient ischemic attack, Secondary prevention, Disease management

## Abstract

**Background:**

Patients with ischaemic stroke or transient ischaemic attack (TIA) are at high risk of future cardiovascular events. Despite compelling evidence about the efficacy of secondary prevention, a substantial gap exists between risk factor management in real life and that recommended by international guidelines. Moreover, stroke is a leading cause of disability and morbidity which partly emerges from post-stroke complications.

**Methods/design:**

We designed a block-randomised (2:1 ratio) open pragmatic trial [NCT02156778] with blinded outcome assessment comparing STROKE-CARD to usual post-stroke-patient care. STROKE-CARD is a multifaceted post-stroke disease management program with the objective of reducing recurrent cardiovascular events and improving quality of life in ischaemic stroke and TIA-patients. It combines intensified multi-domain secondary prevention, systematic detection and treatment of post-stroke complications, and patient self-empowerment. Enrolment of 2160 patients with acute ischaemic stroke or TIA (ABCD2-Score ≥ 3) is planned at two study centres in Austria. The co-primary efficacy endpoints are (i) the composite of major recurrent cardiovascular events (nonfatal stroke, nonfatal myocardial infarction, and vascular death) occurring within 12 months after the index event and (ii) one-year health-related quality-of-life measured with the European Quality of Life-5 Dimensions (EQ-5D-3 L) questionaire. Secondary endpoints include all-cause mortality, functional outcome, and target-level achievement in risk factor management.

**Discussion:**

This trial will provide evidence on whether the pragmatic post-stroke intervention program STROKE-CARD can help prevent cardiovascular events and improve quality-of-life within the setting of a high-quality acute stroke care system. In case of success, STROKE-CARD may be implemented in daily clinical routine and serve as a model for other disease management initiatives.

**Trial registration:**

ClinicalTrials.gov: NCT02156778. (June 5, 2014, retrospectively registered).

**Electronic supplementary material:**

The online version of this article (10.1186/s12883-018-1185-2) contains supplementary material, which is available to authorized users.

## Background

Stroke is the second leading cause of death and one of the leading contributors to disability worldwide [1, 2]. While the age-standardised incidence of stroke is decreasing in high-income countries due to improved health care services and primary prevention, the absolute number of stroke patients is still on the rise, mainly based on continuous population aging and growth [3]. Stroke survivors represent a population-segment particularly vulnerable to further cerebro- and cardiovascular events. Apart from persistent deficits, potentially avoidable medium- and long-term post-stroke complications are significant contributors to functional impairment and an appealing target for concerted interventions.

The risk of stroke recurrence is high at up to 10% within one year and more than 25% within 5 years [4]. Recurrent strokes account for about one fifth of all strokes in state-wide registries [5], have a worse clinical outcome, have a higher fatality rate, and cause higher healthcare costs than first-ever strokes [6].

More than 90% of the global stroke burden is attributable to modifiable risk factors, including behavioural and metabolic factors [7, 8] and most patients with acute ischaemic stroke have one or more un- or insufficiently controlled risk factors - a condition recently termed “preventable stroke” [9]. Vice versa, current evidence-based secondary prevention strategies combining behavioural and pharmacological interventions were estimated to reduce the risk for recurrent vascular events by more than 80% [10]. In real life, however, prevention goals and target levels of risk factors are rarely achieved [11, 12]. Furthermore, discontinuation of prescribed medications represents a major challenge in patient management, occurring in roughly one-third of ischaemic stroke patients within the first year of hospital discharge [13].

Multimodal and multi-disciplinary interventions within the framework of structured disease-management programs have been shown to improve quality of care and outcomes in patients with various chronic diseases other than stroke [14, 15]. Also, digital health care interventions are a promising novel tool to improve individualised risk factor management [16].

To address the substantial gap between risk factor management in real life and that recommended by international guidelines, we designed the STROKE-CARD program, which is a multifaceted pragmatic post-stroke disease management program combining intensified multi-domain secondary prevention, detection and treatment of post-stroke complications, and patient self-empowerment. In a pragmatic trial, we compare STROKE-CARD to usual care in its ability to reduce rates of recurrent cardiovascular events and improve quality-of-life in ischaemic stroke and TIA-patients.

## Methods/design

### Trial objectives

In this trial, we test the hypothesis that the pragmatic disease-management program STROKE-CARD designed for ischaemic stroke and TIA patients is capable of (a) preventing recurrent cardiovascular events by optimised guideline-compliant secondary prevention and target level achievement and (b) ameliorating health-related quality of life (QoL) and patient-wellbeing by early detection and consequent treatment of post-stroke complications.

### Study design and centres

STROKE-CARD is a randomised, controlled, open interventional phase III trial with blinded outcome assessment [ClinicalTrials.gov NCT02156778] conducted in Austria. It initially started as a single-centre study in January 2014 and was extended to a second centre in December 2014. It compares two standards of post-stroke patient care, both complying with the current state-of-the-art. The trial does not involve experimental interventions but compares usual with intensified care as specified below. Primary outcome events are adjudicated based on pre-specified diagnostic criteria blinded to treatment allocation.

The two trial centres are located at the Departments of Neurology at the Innsbruck University Hospital (IUH) and the Hospital St. John of God in Vienna. IUH serves as the comprehensive stroke unit for the entire federal state of Tyrol (catchment area ≈ 1 Mio) and as a primary stroke unit for the city of Innsbruck and 65 surrounding suburban communities with approximately 0.3 Mio inhabitants. Patients from this exclusive catchment area represent an unselected cohort of ischaemic stroke and TIA patients [5]. The Hospital St. John of God Vienna houses one of the four comprehensive stroke units in Vienna. The Viennese stroke network includes additional six primary stroke units and serves the city of Vienna with a catchment area of more than 1.8 Mio inhabitants.

### Study population

The study population comprises consecutive patients with acute ischaemic stroke or TIA (ABCD2-Score ≥ 3) [17] admitted to the two study centres. Patients are eligible for inclusion irrespective of whether the index event was a first or recurrent event. Ischaemic stroke is ascertained using the American Heart Association criteria based on clinical and imaging features [18].

All patients with ischaemic stroke or TIA admitted to the study centres are screened for potential inclusion in the STROKE-CARD trial during the first days of their hospital stay and in most instances included before discharge after signing an informed consent. Further details on the inclusion and exclusion criteria are provided in Table 1.

**Table 1:** STROKE-CARD inclusion and exclusion criteria

Inclusion criteria	Exclusion criteria
Patients with acute ischaemic stroke or TIA (ABCD2 Score ≥ 3 points) Age ≥ 18 years Signed informed consent	Patients living outside the catchment area Malignancy or other severe disease with life-expectancy less than the expected duration of the trial Drug addiction or severe alcohol abuse Patients with permanent severe disability and low perspectives for successful rehabilitation (mRS [19] = 5 at discharge)

*TIA* transient ischaemic attack, *mRS* modified Rankin Scale [19]

### Randomisation and follow-up

**Fig. 1:**
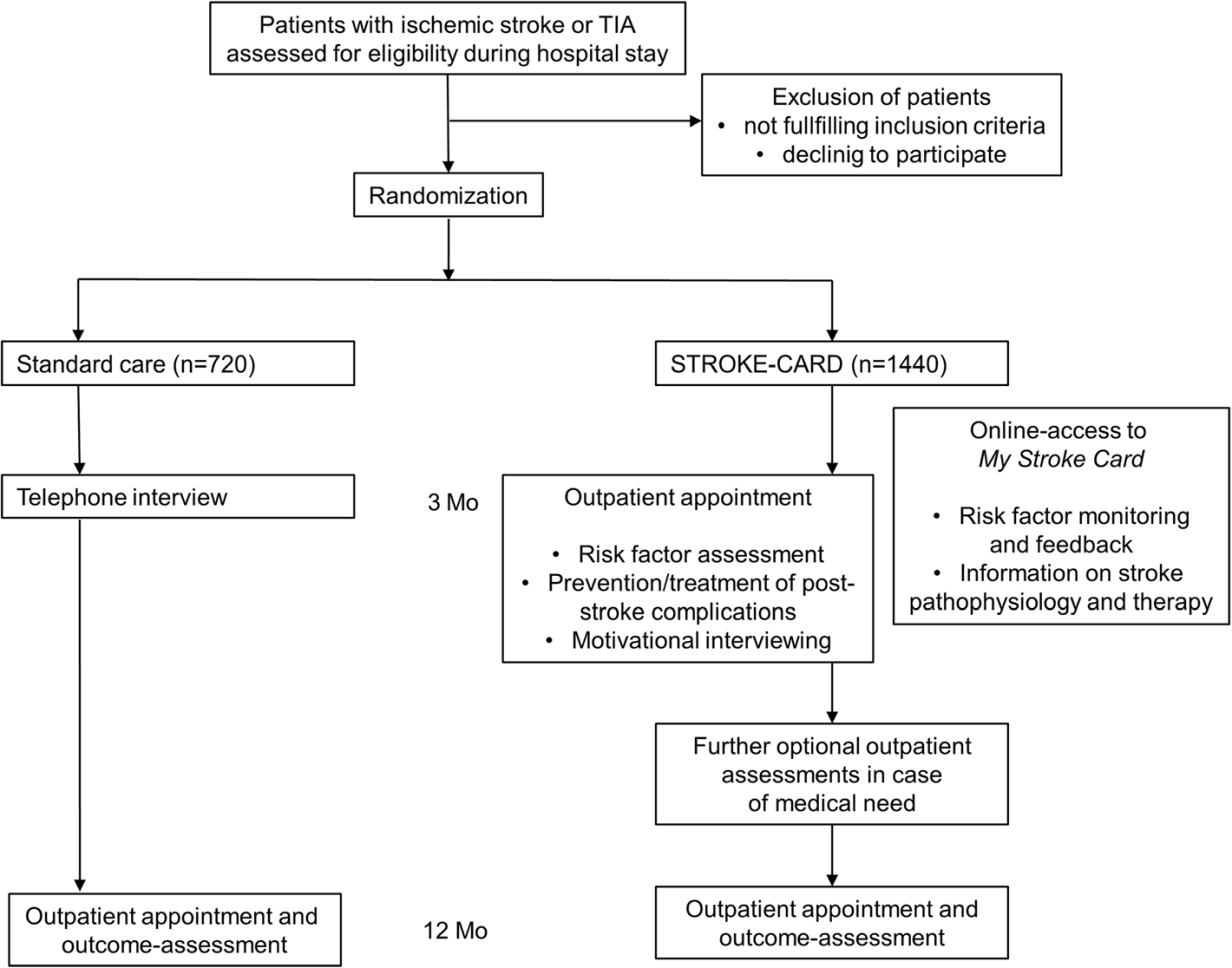
Patient flow and allocation

We block-randomise patients in a 2:1 ratio to receive the STROKE-CARD program or standard care on the basis of the exact date and time of the qualifying event, with the durations of the blocks ranging from 4 to 8 weeks. The target sample size is 2160 patients overall, 1440 patients in the STROKE-CARD group, and 720 in the control group (Fig. 1). We strive for inclusion of about 500 patients per year. Patients are followed-up for 12 months, but we also obtain consent from the patients to contact them later on and extend follow-up for future research projects. Patients who do not adhere to the extended standard care program, especially do not attend the 3-month assessment, are followed in the same way as adherent patients for primary and secondary outcome events.

### Intervention

#### Standard care

In the control arm, patients are managed according to the usual stroke care protocol [5] of the study centres which includes: (a) detailed patient counselling and education about stroke pathophysiology, risk factor management, life style improvement and medication compliance by a stroke specialist (involving the next of kin and caregivers if appropriate); (b) provision of the complimentary book “After a stroke” [20] dealing with all aspects of stroke care; (c) individual in-hospital advice by a dietitian for patients with diabetes, severe dyslipidaemia, and obesity, and smoking cessation support for heavy smokers; (d) provision of standardised information materials (e.g. for oral anticoagulation therapy); and (e) provision at discharge of detailed medical reports for the general practitioner as well as the patient containing target levels for risk factor management. Post-discharge risk-factor-management is performed by the patient’s general practitioner. Only selected high-risk patients are seen in the centres’ outpatient clinics. A standardised 3-month telephone interview by a study nurse for assessment of functional outcome (mRS, Barthel Index, nursing allowance, life situation, support services, medication) is part of the country-wide quality program but has no interventional character [5]. As part of the STROKE-CARD protocol, all patients undergo a comprehensive standardised clinical visit 12 months after recruitment.

#### Stroke-CARD

In addition to the procedures listed above, the intervention-arm includes two additional interventions:

- a three-month outpatient appointment with standardised re-assessment of all risk factors and re-evaluation of stroke aetiology, screening for post-stroke complications (e.g. spasticity, foot-drop, fatigue, pain, incontinence, communication problems, depression and anxiety, impaired cognition, seizures, fatigue, syncope, falls, fractures etc.), other health problems and residual deficits, estimation of the patient’s demand for nursing services and the patient’s adherence to drug prescriptions. All this information is used to optimise secondary prevention [21–23] with best possible achievement of target levels, to refine rehabilitation and treatment goals, and to manage post-stroke complications. Further details on the target levels are listed in Table 2. Patient assessment and counselling is done by a multidisciplinary team of physicians, nurses, physiotherapists, occupational and speech therapists. Treatment decisions are determined by experienced stroke neurologists. The overall visit takes about 3–4 h. The general practitioner receives a detailed medical report with precise instructions and treatment goals. Additional 6-month and 9-month visits are performed exceptionally (target < 5% of cases) at the discretion of the study team in case of medical need, complications requiring follow-up, or very poor risk factor control.
- Patients are offered access to an interactive password-protected web-based patient portal called “**My Strokecard**” [https://ches.tirol-kliniken.at/cms/]. If patients are unable to use a computer, caregivers are asked to assist with the usage of “My Strokecard”. The patient-portal has been designed building on existing software for electronic patient-reported outcome monitoring [24] and contains three components: (i) An adapted version of the ‘post-stroke checklist’ for ascertainment of post-stroke complications is available for screening purposes at any time and pop up automatically three weeks before the three- and twelve-month outpatient appointment, so that the readout of the questionnaires is already available before the medical visit. (ii) A self-administered internet-based tool for risk factor monitoring and patient self-empowerment (i.e. blood pressure, body weight, nicotine consumption, physical activity, LDL-C and HbA1c in case of diabetes) automatically generates feedback on target level achievement [tabular protocols and simple graphs visualising the current stage and time trends for the patient and GP, ***see*** Additional file 1]. (iii) Extensive information and educational materials on stroke pathophysiology, symptoms and risk factor management are provided.

**Table 2:** Target levels and interventions for risk factors at the 3-month risk factor assessment

Condition	Target	Intervention to achieve the targets
Hypertension	BP < 140/90 mmHg BP < 130/85 mmHg in patients with diabetes, renal impairment or small-vessel disease	Adjustment of anti-hypertensive medication Information on supportive lifestyle changes Written recommendations to and intensified management by the GP
Dyslipidaemia	LDL-C < 100 mg/dL LDL-C < 70 mg/dL in very high-risk patients	Adjustment of statin dosage Prescription of ezetimibe additionally to high-dose statins (according to guidelines) Involvement of a lipid-clinic and prescription of PCSK9 inhibitors (according to guidelines) Individualised recommendations by a dietitian
Diabetes	HbA_1c_ < 7%	Re-evaluation of the therapeutic regime by a diabetes-specialist Additional management by the GP, re-instruction in correct administration of injectable pharmacological agents Individualised recommendations by a dietitian
Smoking	Nicotine abstinence	Motivational interviewing and counselling Provision of informational material Involvement of a psychiatric specialist for behavioural or pharmacological therapy
Physical inactivity	Physical activity of moderate to vigorous intensity with an average of 40 min at least 3 times per week	Motivational interviewing Provision of informational material
Non-adherence to drug prescriptions	Adherence to drug prescription (proportion of days covered ≥90%)	Motivational interviewing with information on indication & therapeutic effect of current medication Simplification of drug-regimes Home support by nurses and/or relatives
Poor “stroke-knowledge” of patients & family-members or caregivers		Information on stroke pathophysiology & individual stroke mechanism Provision of a book and standardised information material
Post-stroke complications^a^ & poor functional outcome	Improvement of QoL	Individualised treatment & prevention of post-stroke complications Provision of further outpatient or inpatient rehabilitation Re-assessment on nursing demands, social integration & outpatient care Involvement of a social worker

*BP* blood pressure, *GP* general practitioner, *LDL-C* low-density lipoprotein cholesterol, *PCSK9* proprotein convertase subtilisin/kexin type 9, *HbA*_*1c*_ glycated haemoglobin, *QoL* quality of life

^a^spasticity, pain, risk of falling, post stroke-depression (PSD), fatigue, anxiety, dysphagia, social deprivation, post-stroke dementia

**Table 3:** Trials on multimodal secondary prevention strategies in ischaemic stroke or TIA-patients

Study, Country	Y	Inclusion criteria	n	Age	M (%)	Intervention type/model	FU (Mo)	Outcome measures	Significant results
PRAISE, USA [42]	2014	Ischaemic stroke, TIA < 5 years, age > 40 y	600	63 ± 11	40	Education & self-management (peer-led), 6 weekly workshops	6	Cholesterol, BP, antithrombotics use	BP-lowering
ICARUSS, Australia [43]	2009	Ischaemic stroke, haemorrhagic stroke, TIA, age > 20 y	233	66 ± 13	54	Education & pre-arranged visits at the GP’s at 2 weeks, 3, 6, 9, 12 Mo. Telephone assessment prior to each visit	12	RF-modification, disability, QoL, cognitive function, ADLs	Cholesterol-, BP-lowering, exercise, disability, QoL
Hornnes et al., Denmark [44]	2011	Ischaemic stroke or TIA, all age-groups	349	69 ± 12	45	Pre-discharge or outpatient appointment, nurse-led home visits at 1, 4, 7, 10 Mo	12	BP after one year	BP-lowering
INSPiRE-TMS, Germany [51]	2013	TIA, minor stroke, age > 18 y	Target = 2082	N/A	N/A	RF-management & support program, up to 8 assessments	24	Stroke, ACS, cardiovascular death, RF-control, mortality, hospital admissions	ongoing
SMART study, China [45]	2014	Ischaemic stroke, TIA related to atherosclerosis	3821	61 ± 12	68	Medication & lifestyle advice, education (computer software)	12	Adherence to drugs, stroke, ACS, all-cause death	better adherence to statins
STANDFIRM, Australia [46]	2017	Ischaemic stroke, haemorrhagic stroke, TIA, age > 18 y	563	70	N/A	Community-based intervention, evidence-based care plan, 3 education sessions, 2 telephone assessments	24	Targets for cardiometabolic factors	cholesterol levels
COMPASS, USA [52]	2017	Ischaemic stroke, haemorrhagic stroke, TIA, age > 18 y	Target = 6000	N/A	N/A	holistic approach integrating medical & community resources, clinical visit after 14 days, 4 telephone assessments	3	Functional status, Qol, cognitive function, hospitalisations, caregiver measures	ongoing
SUCCEED, USA [53]	2017	Ischaemic stroke, TIA, haemorrhagic stroke, hypertension, age > 40 y	Target = 516	N/A	N/A	3 clinic visits, 3 home visits, & telephone coordination by community health worker, self-coordination program	36	BP, RF-control, medication adherence, cost-effectivness	ongoing
NAILED, Sweden [47]	2015	Ischaemic stroke, haemorrhagic stroke, TIA, all age-groups	537	71 ± 11	57	Nurse-led, telephone-based follow-up, medication adjustment	12	BP, LDL-C, RF-control	cholesterol-, BP-lowering
Kono et al., Japan [48]	2013	Ischaemic stroke (mRS 0–1), non-cardio-embolic origin	70	64	68	Lifestyle intervention program with counselling at BL, 3, 6 Mo, exercise training (2x/week) for 24 weeks	36	Stroke or cardiac death, hospitalisation due to stroke recurrence, MI, AP or pAD, RF-control	vascular events, physical activity BP-lowering, salt intake.
McAlister et al., Canada [49]	2014	Ischemic stroke, TIA, slight or no disability	275	68	63	Pharmacist-led or a nurse-led case manager intervention with 6 monthly visits	12	BP and lipid control, FRS and CDLEM	cholesterol-, BP-lowering, global vascular risk
STROKE-CARD, Austria	2017	Ischaemic stroke (mRS 0–4), TIA (ABCD2-Score ≥ 3); age > 18 y	Target = 2170	N/A	N/A	3 Mo clinical visit with RF-assessment, online RF-monitoring	12	Major cardiovascular event, vascular death, QoL	ongoing

*ACS* acute coronary syndrome, *ADLs* activities of daily living, *AP* angina pectoris, *BL* baseline, *BP* blood pressure, *CDLEM* Cardiovascular Disease Life Expectancy Model, *FRS* Framingham Risk Score, *FU* follow up, *GP* general practitioner, *M* percentage of male participants, *Mo* month, *MI* myocardial infarction, *N/A* not available, *pAD* peripheral artery disease, *QoL* quality-of-life, *RF* risk factor, *SBP* systolic blood pressure, *TIA* transient ischaemic attack

During the in-hospital phase, clinical care does not differ between the two groups, with the exception of the training for the use of “My Stroke Card” in the extended care group before discharge.

There will be (i) a continuous monitoring of different benchmarks, including the duration of hospital stay and access to rehabilitation facilities to ensure that the two groups do not receive differential attention and to minimise surveillance biases, and (ii) a monitoring of trial progress by a data monitoring board.

### Outcome

#### Primary outcomes (Co-primary endpoint)

- ▪ Incidence of major cardiovascular events defined as nonfatal stroke (ischaemic or haemorrhagic), nonfatal myocardial infarction (including acute coronary syndrome requiring emergency vascularisation), and vascular death (i.e. sudden cardiac death and death from acute myocardial infarction, ischaemic or haemorrhagic stroke, heart failure, cardiovascular procedures, pulmonary embolism, or peripheral artery disease) within one year of the index event.
- ▪ Health-related QoL assessed by the European Quality of Life-5 Dimensions (EQ-5D-3 L) overall health utility score one year after the index event.

#### Secondary outcomes

- ▪ Recurrent ischaemic or haemorrhagic stroke and TIA (defined as transient neurological deficit < 24 h and absence of DWI positive lesions on MRI) within one year of the index-event.
- ▪ All-cause mortality at 12 months.
- ▪ Favorable functional outcome (mRS ≤2 and change in mRS) at one year and at three months.
- ▪ Individual components of the EQ-5D-3 L questionnaire, i.e. mobility, self-care, usual activities, pain and discomfort, anxiety and depression after one year.
- ▪ Achievement of predefined target levels in secondary prevention 12 months after the index event: BP < 140/90 mmHg [< 130/85 mmHg in selected patients], target HbA_1c_ in patients with diabetes [mainly < 7.0%, less stringent targets in elderly], nicotine abstinence, LDL-C < 100 mg/dL [LDL-C < 70 mg/dL in high-risk patients] [23], physical activity with an average of 40 min at least 3 times per week, platelet inhibitor or anticoagulation according to ischaemic stroke aetiology [in case of oral anticoagulation with vitamin K antagonists: INR 2–3, time in therapeutic range (TTR) > 70%], statins except for patients with ischaemic strokes of non-atherosclerotic origin and no evidence of atherosclerosis (e.g. vessel dissection), medication adherence [proportion of days covered (PDC) ≥ 90%] [25]. The latter criterion focuses on statins, platelet inhibitors other than aspirin (which is typically purchased over the counter), antihypertensive medication, oral anti-diabetic drugs, and anticoagulation.

### Clinical assessments and study protocols

The protocol uses validated and field-proven forms, scales and questionnaires. All the study team members are highly experienced and NIHSS- and mRS-certified.

The clinical baseline-assessment (entire study population) and the outpatient appointment (3-month intervention [intervention arm only] and 12-month outcome assessment [entire study population]) will be performed by trained health care professionals of the multidisciplinary study team composed of experienced stroke neurologists, PhD-students, physiotherapists, and stroke nurses. Data are collected prospectively according to pre-specified protocols and questionnaires with electronic data entry and storage and undergo subsequent quality checks and validation (e.g. by hospital records and feedback from the GP). All hospitals in the survey area dispose of electronic records, to which study investigators were granted access via protected data links according to the patients’ informed consent.

The **baseline assessment** includes detailed documentation of the following components: ischaemic stroke aetiology [TOAST-criteria [26], Causative Classification of Stroke [27]], stroke severity [NIHSS [28] on admission and discharge], clinical syndrome [stroke/TIA symptoms, vessel territories], acute stroke therapy and recanalization procedures, pre-stroke disability and disability on admission [mRS], prior cardiovascular events and comorbidities, cardiovascular risk factors (hypertension, dyslipidaemia, smoking-status [current, former, never] and burden [pack-years, Fagerstroem questionnaire [29]], alcohol consumption [quantity, patterns, type of beverages], diabetes [ADA criteria] [30], atrial fibrillation, physical activity [Baecke Physical Activity Questionnaire] [31]), family history of cardiovascular disease, previous and current medication, cognitive function [Mini-Mental State Examination [32] and Montreal Cognitive Assessment [33]], depression [Beck Depression Inventar, anxiety [Hospital Anxiety and Depression Scale [34]], fatigue [Fatigue Severity Scale [35]], health related quality of life [European Quality of Life-5 Dimensions EQ5D-3 L [36]], anthropometric measures [body mass index, waist–to hip ratio], repeated blood pressure recordings [acute setting and before discharge], ankle-brachial index, atherosclerosis of the carotid arteries [intima-media thickness, plaque burden], [37] pulse wave velocity [Complior® system], and routine laboratory measures [including lipid profile, renal function, liver function, thyroid function, cardiac markers, differential blood count, coagulation diagnostics, and inflammation markers].

The **3- and 12-month visits** involve a neurological evaluation with documentation of clinical deficits and functional outcome [NIHSS, mRS, and Barthel Index [38]], routine laboratory examination and assessment of recurrent cardiovascular events, hospital stays and procedures [interventional and surgical], falls and fractures, all incident diseases and morbidities [including bleeding events], current medication, adherence to prescribed drugs, and risk factor control. Cognitive function, depression, anxiety, fatigue, and QoL all are re-evaluated [see baseline assessment]. Carotid ultrasound [see baseline protocol] is obligatorily repeated during the 12-month visit and, if clinically indicated, also after three months.

Blood and urine samples are drawn after an overnight fast and at least 12-h abstinence from smoking [during the hospital stay and 3 and 12 months thereafter], immediately processed, used for routine testing, and stored in a biobank [plasma, serum, whole blood, and urine]. Samples are stored at − 80 °C in compliance with the OECD guidelines and Austrian Bioethics Commission recommendations [39].

### Sample size

Sample size calculations for this trial were based on the primary composite cardiovascular disease endpoint. Assuming a 1-year cumulative risk of 15% in the usual care group, 2045 subjects are required to detect a 5% absolute risk reduction (α = 0.05) with a 90% power.

Expecting an attrition rate of 15% (7.5% dropout rate [withdrawal of consent] and a loss-to-follow-up of 7.5%), we aimed to include a total of 2400 patients. Between January 2014 and June 2017, 1866 patients were recruited, with an attrition rate substantially lower than expected (< 5.0% as opposed to the anticipated 15%). Accordingly, the data monitoring board recommended revision of the recruitment goal from 2400 to 2160 patients. A sample size of 2160 patients provides the trial with a 90% power to detect a difference of 0.03 points on the EQ-5D-3 L overall health utility score (co-primary efficacy endpoint) assuming a standard deviation of 0.2.

### Statistical analyses

The primary efficacy analysis is based on time from hospital discharge to first occurrence of primary composite end-point as defined above. It is an unadjusted survival analysis according to the intention-to-treat principle with use of the log-rank test. Hazard ratios and 95% confidence intervals will be estimated using Cox models with the assumption of proportional hazards checked by means of Schoenfeld residuals. Prespecified sensitivity analyses include adjusted models and a per-protocol analysis. Consistency of intervention effects in four prespecified subgroups (men and women, age groups, type of index event, and patients with or without regular use of My StrokeCard) will be assessed by means of test for interaction. The continuous efficacy endpoint QoL will be analysed with linear regression models.

Owing to the fact that our study attempts to encourage patients to adhere to current evidence-based prevention guidelines and does not include experimental treatment concepts, we do not perform a formal safety analysis. We however analyze whether there are any differences in the occurrence of potential side effects, risks and consequences of intensified secondary prevention in both study arms including (a) major bleeding [40], (b), syncopes and fractures, (c) laboratory abnormalities (ALT, CK, GFR), and (d) muscle-related outcomes. No formal interim analysis will be performed. All reported *P* values are two-sided.

## Discussion

The risk of stroke recurrence within one year after ischaemic stroke or TIA is high and can effectively be reduced by effective guideline-compliant secondary prevention. Vice versa, poor adherence to guidelines leads to preventable recurrent events, worse outcome and higher health care costs. Post-stroke complications are frequent, often remain unrecognised and contribute to stroke disability and poor QoL.

### The STROKE-CARD concept

STROKE-CARD is the first comprehensive pragmatic post-stroke disease management in Austria and, to our knowledge, one of the largest interventions of its kind worldwide. STROKE-CARD is addressing a real-life-cohort of ischaemic stroke- and TIA-patients with the primary objectives of enhancing adherence to prevention guidelines and thereby lowering rates of recurrent vascular events and post-stroke complications, and improving functional outcome and QoL. Whereas disease management programs traditionally rely on expert opinion, our initiative strives for a rigorous scientific evaluation involving outcome and health economy analyses. It pursues the concept that optimal acute stroke care extends to a thorough three-month assessment and individualised counselling. We employ a multidisciplinary but lean and cheap intervention leveraging contemporary e-technology and encouraging patient self-empowerment with the prospect of a widespread implementation in case of success. Access to Internet and social media is now broadly available and STROKE-CARD offers web-based educational patient-operated tools for risk factor to record and manage risk factors combined with linkage of patient data to medical caregivers. The STROKE-CARD concept was presented as an abstract at the European Stroke Organisation Conference 2018 [41].

### Other post-stroke disease management programs

Previous trials on multimodal secondary prevention strategies in ischaemic stroke or TIA-patients have shown variable improvements in risk factor control or medication adherence but most were not designed and powered to analyse potential effects recurrent cardiovascular disease and stroke events. An overview on trials focusing on multimodal secondary stroke prevention is provided in Table 3. Limitations shared by these trials include the usually limited sample size (443 median, IQR 347.3) [42–50] and the common focus on TIA or minor stroke patients only [48, 49, 51].

Only one large-scale study has been completed so far [45] and enrolled more than 3800 ischaemic stroke and TIA-patients in 47 hospitals in China. In spite of a better adherence to statin-medications no difference in the hard clinical end points after one year of follow-up could be demonstrated.

Two ongoing studies deserve special consideration: (i) The Comprehensive Post-Acute Stroke Service (COMPASS) study, [52] a cluster-randomised trial involving 40 hospitals in North Carolina, plans enrolment of 6000 ischaemic and haemorrhagic stroke patients and investigates potential effects of the COMPASS care model (telephone follow-up, one clinic visit within two weeks after discharge, and an individualised patient electronic care plan) on the patient-reported 90-day functional outcome (primary endpoint), hospital re-admission rates, and 90-day mortality. (ii) The Intensified Secondary Prevention intending a Reduction of Recurrent Events in TIA and Minor Stroke patients (INSPiRE-TMS) study, [51] from Germany intends to recruit 2082 patients with minor stroke or TIA, provides up to eight appointments in outpatient clinics and focuses on two-year recurrent vascular events or vascular death.

Type and intensity of intervention vary widely among the different trials. Several programs envisage nurse-led home visits or telephone-based assessments for risk factor control [44, 47, 50]. The ICARUSS-strategy [43] in Australia trusts on several pre-arranged GP visits whereas the STANDFIRM-trial (*n* = 500), [46] a community-based intervention, consists of educational sessions, multiple follow-up assessments, and as telephone interviews conducted by study nurses and general practices. A recent intervention [49] has achieved a substantial and sustained (six months) reduction in global vascular risk (Framingham Risk Score, Cardiovascular Disease Life Expectancy Model) by non-physician provider case management. Digital health interventions (e.g. telemedicine, web-based strategies, email and- mobile phone-reminders) improved risk factor control in vascular diseases other than stroke [16].

### Strengths and limitations

Key and in part unique features of STROKE-CARD are its comprehensive focus on both recurrent vascular events (risk factor control and compliance) and post-stroke complications and QoL, its lean and easily applicable intervention, and the inclusion of moderate and severe ischaemic stroke patients (all except those with permanent severe disability).

The primary analysis may be viewed as conservative in two respects: [1] It relies on a short one-year follow-up whereas potential effects are expected to increase over time. Extension of follow-up is a decided goal and fund raising is in progress [2]. Our program is realised within the framework of a highly developed stroke care setting and bears the challenge of a strong comparator group with high-quality management of ischaemic stroke/TIA patients. Although findings of our trial are not easily extrapolated to less developed stroke systems, the potential benefit of the intervention is presumably even higher on the low-quality stroke care background.

Our program is focused on ischaemic stroke but may serve as a model for other diseases sensitive to post-discharge management.

In case of success, the next steps would be a refinement of STROKE-CARD components and identification of subgroups with a health benefit and cost effectiveness in order to end up with a practicable tool for broad clinical implementation in the whole of Tyrol, Austria and beyond.

## Additional file

Additional file 1: Pragmatic Trial of Multifaceted Intervention (STROKE-CARD care) to Reduce Cardiovascular Risk and Improve Quality-of-Life after Ischaemic Stroke and Transient Ischaemic Attack –Study protocol. Excerpt of the interactive website “My Strokecard”. (DOCX 389 kb)

## Abbreviations

ACS: Acute coronary syndrome
ADLs: Activities of daily living
ALT: Alanine aminotransferase
AP: Angina pectoris
BL: Baseline
BP: Blood pressure
CDLEM: Cardiovascular Disease Life Expectancy Model
CK: Creatine kinase
DWI: Diffusion weighted imaging
EQ-5D-3L: European Quality of Life-5 Dimensions
FRS: Framingham Risk Score
FU: Follow up
GFR: Glomerular filtration rate
GP: General practitioner
HbA1c: Glycated haemoglobin
IUH: Innsbruck University Hospital
LDL-C: Iow-density lipoprotein cholesterol
MI: Myocardial infarction
Mo: Month
MRI: Magnet resonance imaging
mRS: Modified Rankin Scale
N/A: Not available
NIHSS: National institute of health stroke scale
pAD: Peripheral artery disease
PCSK9: proprotein convertase subtilisin/kexin type 9
PDS: Post stroke-depression
QoL: Quality of life
RF: Risk factor
SBP: Systolic blood pressure
TIA: Transient ischaemic attack
TOAST: Trial of Org 10,172 in Acute Stroke Treatment
