# Supplementary material for: Pragmatic trial of multifaceted intervention (STROKE-CARD care) to reduce cardiovascular risk and improve quality-of-life after ischaemic stroke and transient ischaemic attack –study protocol
Source: BMC Neurol. 2018 Nov 6;18:187. doi: 10.1186/s12883-018-1185-2 (PMC6219064; doi:10.1186/s12883-018-1185-2)
Supplement: Supplementary file 1 — Pragmatic Trial of Multifaceted Intervention (STROKE-CARD care) to Reduce Cardiovascular Risk and Improve Quality-of-Life after Ischaemic Stroke and Transient Ischaemic Attack –Study protocol. Excerpt of the interactive website “My Strokecard”. (DOCX 389 kb) [file 12883_2018_1185_MOESM1_ESM.docx]

**Pragmatic Trial of Multifaceted Intervention (STROKE-CARD care) to Reduce Cardiovascular Risk and Improve Quality-of-Life after Ischaemic Stroke and Transient Ischaemic Attack –Study protocol.**

**Additional file 1**

**Figure S1.** Excerpt of the interactive website “My Strokecard”


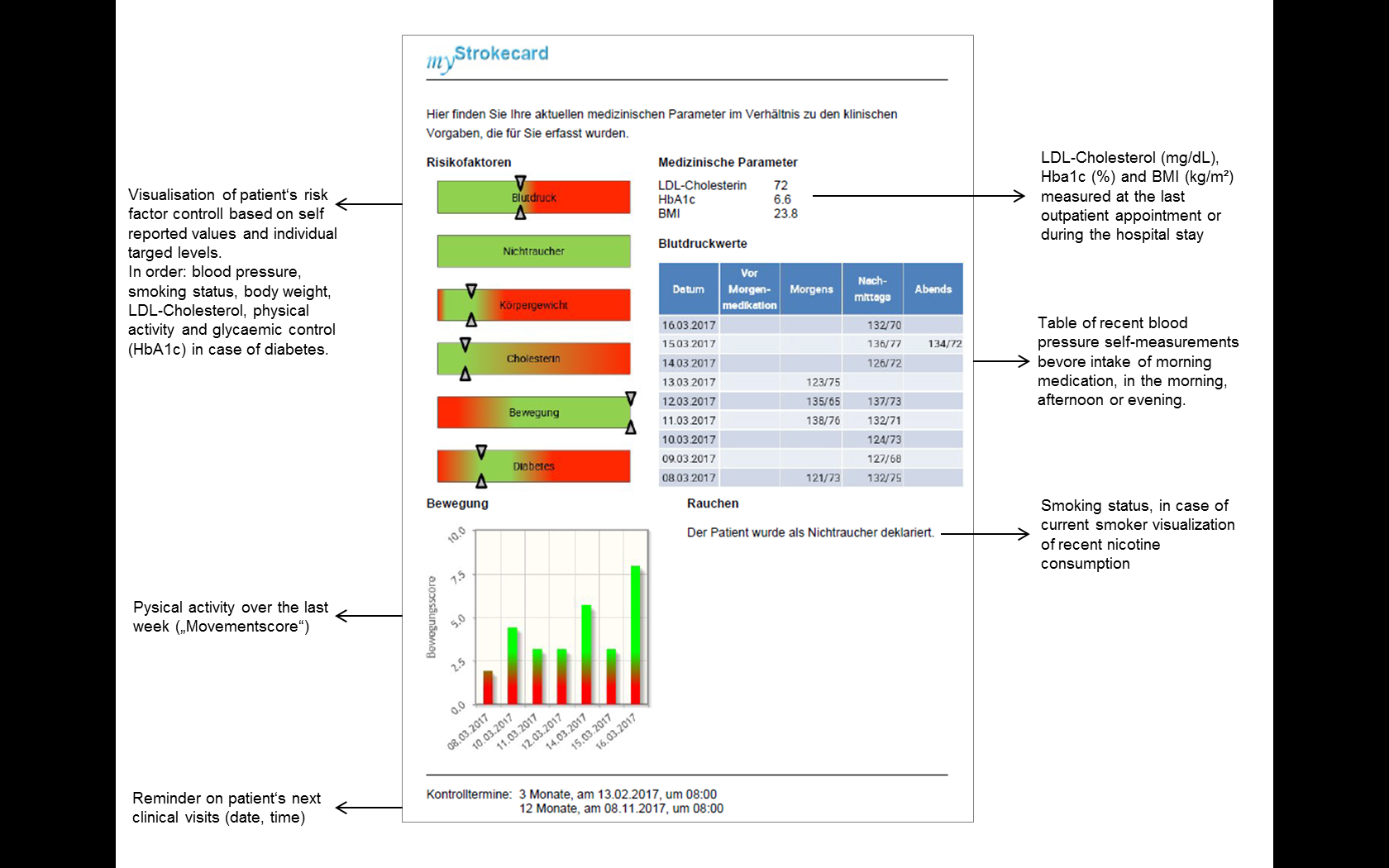


LDL: low density lipoprotein; BMI: body mass index
